# Supplementary material for: Breast Self-Examination Practice and Its Determinants among Women in Indonesia: A Systematic Review, Meta-Analysis, and Meta-Regression
Source: Diagnostics (Basel). 2023 Aug 2;13(15):2577. doi: 10.3390/diagnostics13152577 (PMC10416892; doi:10.3390/diagnostics13152577)
Supplement: Supplementary file 1 [file diagnostics-13-02577-s001.zip › diagnostics-2435081-Figure S1.pdf]

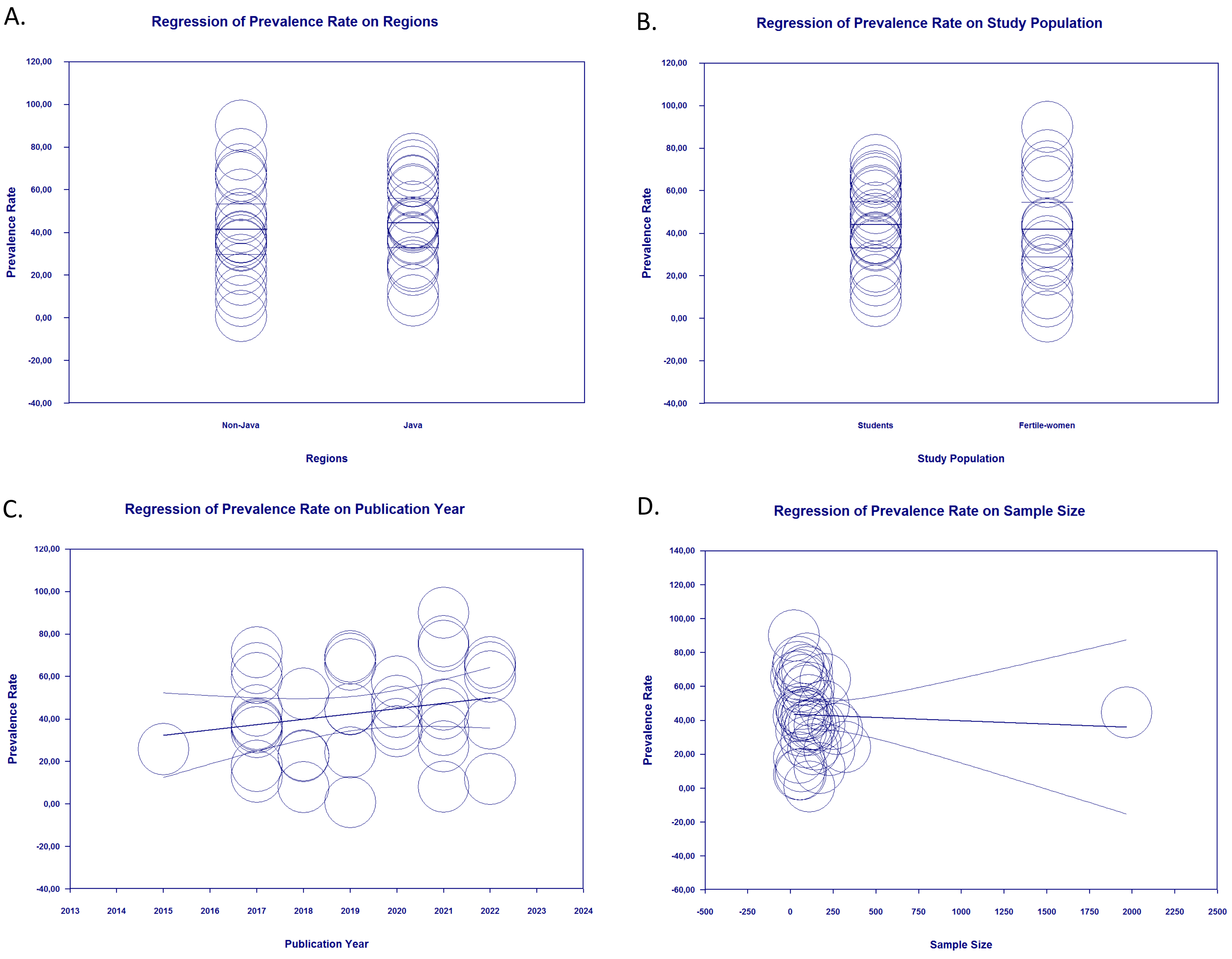
A.
Regression of Prevalence Rate on Regions
Prevalence Rate
120,00
100,00
80,00
60,00
40,00
20,00
0,00
-20,00
-40,00
Non-Java
Java
Regions
B.
Regression of Prevalence Rate on Study Population
Prevalence Rate
120,00
100,00
80,00
60,00
40,00
20,00
0,00
-20,00
-40,00
Students
Fertile-women
Study Population
C.
Regression of Prevalence Rate on Publication Year
Prevalence Rate
120,00
100,00
80,00
60,00
40,00
20,00
0,00
-20,00
-40,00
2013
2014
2015
2016
2017
2018
2019
2020
2021
2022
2023
2024
Publication Year
D.
Regression of Prevalence Rate on Sample Size
Prevalence Rate
140,00
120,00
100,00
80,00
60,00
40,00
20,00
0,00
-20,00
-40,00
-60,00
-500
-250
0
250
500
750
1000
1250
1500
1750
2000
2250
2500
Sample Size
