# Supplementary material for: Breast Self-Examination Practice and Its Determinants among Women in Indonesia: A Systematic Review, Meta-Analysis, and Meta-Regression
Source: Diagnostics (Basel). 2023 Aug 2;13(15):2577. doi: 10.3390/diagnostics13152577 (PMC10416892; doi:10.3390/diagnostics13152577)
Supplement: Supplementary file 1 [file diagnostics-13-02577-s001.zip › diagnostics-2435081-Tables S1 and S2.pdf]

Supplementary Table S1. Joanna Briggs Institute critical appraisal checklist for studies reporting prevalence data

**Author, year**	**Question 1**	**Question 2**	**Question 3**	**Question 4**	**Question 5**	**Question 6**	**Question 7**	**Question 8**	**Question 9**	**Overall Appraisal**
Adimuntja NP, et al. 2022	Yes	Yes	Yes	Yes	Yes	Yes	Yes	Yes	Yes	8
Ajeng A, et al. 2017	No	Yes	Yes	Yes	Yes	Yes	Yes	Yes	Yes	8
Amelia C, et al. 2021	Yes	Yes	Yes	Yes	Yes	Yes	Yes	Yes	Yes	9
Angrainy R, 2017	Yes	Yes	Yes	Yes	Yes	Yes	Yes	Yes	Unclear	8
Arafah ABR, et al. 2017	Yes	Yes	Yes	Yes	Yes	Yes	Yes	Yes	Yes	9
Artikasari L, et al. 2021	Yes	Yes	Yes	No	Yes	Yes	Yes	Yes	Unclear	7
Cane PS, et al. 2019	No	Yes	Yes	Yes	Yes	Yes	Yes	Yes	Yes	8
Despitasari L, et al. 2017	No	Yes	Yes	Yes	Yes	Yes	Yes	Yes	Yes	8
Dewi CI, et al. 2017	Yes	Yes	Yes	Yes	Yes	Yes	Yes	Yes	Yes	9
Dewi R, et al. 2021	No	Yes	Yes	Yes	Yes	Yes	Yes	Yes	Yes	8
Dewi TK, et al. 2019	Yes	Yes	Yes	Yes	Yes	Yes	Yes	Yes	Yes	9
Dewi TK, et al. 2022	Yes	Yes	Yes	Yes	Yes	Unclear	Yes	Yes	Yes	8
Fahriani M, et al. 2021	No	Yes	Yes	Yes	Yes	Unclear	Yes	Yes	Yes	7
Fatrin T, et al. 2020	Yes	Yes	Yes	Yes	Yes	Yes	Yes	Yes	Unclear	8
Ginting L, 2019	No	Yes	Yes	Yes	Yes	Yes	Yes	Yes	Yes	8
Hanifah AN, 2015	No	Yes	Yes	Yes	Yes	Yes	Yes	Yes	Yes	8
Herman IVI, et al. 2019	Yes	Yes	Yes	Yes	Yes	Unclear	Yes	Yes	Yes	8
Immawati, et al. 2020	No	Yes	Yes	Yes	Yes	Yes	Yes	Yes	Yes	8
Karnawati PWW, et al. 2022	Yes	Yes	Yes	Yes	Yes	Yes	Yes	Yes	Yes	9
Khairunnissa A, et al. 2017	Yes	Yes	Yes	Yes	Yes	Yes	Yes	Yes	Yes	9
Khotimah S, 2019	Yes	Yes	Yes	Yes	Yes	Yes	Yes	Yes	Yes	9
Kris D, 2019	No	Yes	Yes	Yes	Yes	Yes	Yes	Yes	Yes	8
Kurniawati T, et al. 2021	Yes	Yes	Yes	Yes	Yes	Yes	Yes	Yes	Yes	9
Kusumaningrum TAI, et al. 2018	Yes	Yes	Yes	Yes	Yes	Yes	Yes	Yes	Yes	9
Lula F, et al. 2018	No	Yes	Yes	Yes	Yes	Yes	Yes	Yes	Yes	8
Mularsih S, et al. 2017	Yes	Yes	Yes	Yes	Yes	Yes	Yes	Yes	Yes	9
Puspitasari YD, et al. 2021	Yes	Yes	Yes	Yes	Yes	Yes	Yes	Yes	Yes	9
Riadinata, et al. 2021	Yes	Yes	Yes	Yes	Yes	Yes	Yes	Yes	Yes	9
Sari IG, et al. 2022	No	Yes	Yes	Yes	Yes	Yes	Yes	Yes	Yes	8
Sari RJ, et al. 2017	Yes	Yes	Yes	Yes	Yes	Yes	Yes	Yes	Yes	9
Sari WY, et al. 2022	No	Yes	Yes	Yes	Yes	Yes	Yes	Yes	Yes	8
Sebayang W, et al. 2018	No	Yes	Yes	Yes	Yes	Yes	Yes	Yes	Yes	8
Siboro YK, et al. 2018	Yes	Yes	Yes	Yes	Yes	Yes	Yes	Yes	Unclear	8
Siregar R, 2019	No	Yes	Yes	Yes	Yes	Yes	Yes	Yes	Yes	8
Sunarni N, et al. 2020	No	Yes	Yes	Yes	Yes	Yes	Yes	Yes	Yes	8
Supatmi, 2021	No	Yes	Yes	Yes	Yes	Yes	Yes	Yes	Yes	8
Susanti R, 2017	Yes	Yes	Yes	Yes	Yes	Yes	Yes	Yes	Yes	9
Tuelah G, et al. 2020	Yes	Yes	Yes	Yes	Yes	Yes	Yes	Yes	Yes	9
Wahyuni S, et al. 2020	No	Yes	Yes	Yes	Yes	Yes	Yes	Yes	Yes	8
Wantini NA, et al. 2017	Yes	Yes	Yes	Yes	Yes	Yes	Yes	Yes	Yes	9
Wulandari F, et al. 2017	Yes	Yes	Yes	Yes	Yes	Yes	Yes	Yes	Yes	9
Zulaika C, et al. 2021	Yes	Yes	Yes	Yes	Yes	Yes	Yes	Yes	Yes	9

Question 1. Was the sample frame appropriate to address the target population?
Question 2. Were study participants sampled in an appropriate way?
Question 3. Was the sample size adequate?
Question 4. Were the study subjects and the setting described in detail?
Question 5. Was the data analysis conducted with sufficient coverage of the identified sample?
Question 6. Were valid methods used for the identification of the condition?
Question 7. Was the condition measured in a standard, reliable way for all participants?
Question 8. Was there appropriate statistical analysis?
Question 9. Was the response rate adequate, and if not, was the low response rate managed appropriately?

Intepretation:
1 – 3: Low quality
4 – 6: Moderate quality
7 – 9: High quality

Supplementary Table S2. Results of the meta-regression models for the national prevalence of breast self-examination practice in Indonesia

**Covariate**	**Coefficient**	**95% CI (min)**	**95% CI (max)**	**SE**	***p*-value**
Regions	2.9608	-10.2193	16.1410	6.7247	0.6597
Study population	-2.2602	-15.6472	11.1268	6.8302	0.7407
Publication year	2.5152	-0.9246	5.9550	1.7550	0.1518
Sample size	-0.0039	-0.0263	0.0185	0.0114	0.7352

CI – Confidence Interval, SE – Standard Error
